# Genome sequencing with comprehensive variant calling identifies structural variants and repeat expansions in a large fraction of individuals with ataxia and/or neuromuscular disorders

**DOI:** 10.3389/fneur.2023.1170005

**Published:** 2023-05-18

**Authors:** Marlene Ek, Daniel Nilsson, Martin Engvall, Helena Malmgren, Håkan Thonberg, Maria Pettersson, Britt-Marie Anderlid, Anna Hammarsjö, Hafdis T. Helgadottir, Snjolaug Arnardottir, Karin Naess, Inger Nennesmo, Martin Paucar, Helgi Thor Hjartarson, Rayomand Press, Göran Solders, Thomas Sejersen, Anna Lindstrand, Malin Kvarnung

**Affiliations:** ^1^Department of Molecular Medicine and Surgery, Karolinska Institutet, Stockholm, Sweden; ^2^Department of Clinical Genetics, Karolinska University Hospital, Stockholm, Sweden; ^3^Science for Life Laboratory, Department of Molecular Medicine and Surgery, Karolinska Institutet Science Park, Solna, Sweden; ^4^Karolinska University Hospital, Centre for Inherited Metabolic Diseases, Stockholm, Sweden; ^5^Department of Neurology, Karolinska University Hospital, Stockholm, Sweden; ^6^Department of Medical Biochemistry and Biophysics, Karolinska Institutet, Stockholm, Sweden; ^7^Department of Oncology-Pathology, Karolinska University Hospital, Stockholm, Sweden; ^8^Department of Neuropediatrics, Astrid Lindgren Children's Hospital, Karolinska University Hospital, Stockholm, Sweden; ^9^Department of Clinical Neurophysiology, Karolinska University Hospital, Stockholm, Sweden; ^10^Department of Women's and Children's Health, Karolinska Institutet, Stockholm, Sweden

**Keywords:** neuromuscular disorders, genome sequencing, single nucleotide variant, structural variant, repeat expansion, ataxia

## Abstract

**Introduction:**

Neuromuscular disorders (NMDs) have a heterogeneous etiology. A genetic diagnosis is key to personalized healthcare and access to targeted treatment for the affected individuals.

**Methods:**

In this study, 861 patients with NMDs were analyzed with genome sequencing and comprehensive variant calling including single nucleotide variants, small insertions/deletions (SNVs/INDELs), and structural variants (SVs) in a panel of 895 NMD genes, as well as short tandem repeat expansions (STRs) at 28 loci. In addition, for unsolved cases with an unspecific clinical presentation, the analysis of a panel with OMIM disease genes was added.

**Results:**

In the cohort, 27% (232/861) of the patients harbored pathogenic variants, of which STRs and SVs accounted for one-third of the patients (71/232). The variants were found in 107 different NMD genes. Furthermore, 18 pediatric patients harbored pathogenic variants in non-NMD genes.

**Discussion:**

Our results highlight that for children with unspecific hypotonia, a genome-wide analysis rather than a disease-based gene panel should be considered as a diagnostic approach. More importantly, our results clearly show that it is crucial to include STR- and SV-analyses in the diagnostics of patients with neuromuscular disorders.

## 1. Introduction

Neuromuscular disorders (NMDs) encompass a heterogeneous group of disorders that affect the function of peripheral motor nerves, muscles, or neuromuscular junction. Common symptoms include weakness, hypotonia, muscle atrophy, contractures, sensory disturbances, and symptoms from the autonomic nervous system, with onset varying from prenatal to late adulthood. A wider definition of NMDs includes also spastic paraplegia and ataxias. NMDs are caused by constitutional genetic aberrations and/or non-genetic factors.

The number of genes associated to NMDs is continuously increasing and, as of December 2022, 641 genes were listed in the gene table for NMDs, as hosted by Cohen et al. (1) and Dalil Hamroun and Rivier (2). Single nucleotide variants (SNVs)/small insertions-deletions (INDELs) in these genes are the most prevalent type of pathogenic variants, while other variant types such as short tandem repeat expansions (STRs) and structural variants (SVs) are causative in a subset of NMD patients. Given the heterogeneous etiology of NMDs, a clinical diagnostic workup is often challenging. A precise molecular diagnosis is valuable for predicting clinical course, and it is crucial for genetic counseling including carrier testing, prenatal diagnostics, or preimplantation genetic testing. In addition, it is a prerequisite for getting access to targeted treatments and inclusion in clinical trials for novel therapeutics that are gene- and/or mutation-specific.

Several studies of clinical genetic testing in patients with NMDs, using gene panels based on massive parallel sequencing to detect SNVs, have been published with the diagnostic yield varying between 13 and 53% (3–10). However, with this approach, variants other than exonic SNVs/INDELs would go undetected. Recently, efforts in using genome sequencing and bioinformatic pipelines to detect repeat expansions show that this method has a high sensitivity and specificity with an additional diagnostic yield up to 3% in cohorts of various neurological disorders (11, 12).

In this study, we evaluate the clinical utility of genome sequencing with comprehensive variant calling using pipelines for the detection of SNVs/INDELs, STRs, and SVs in a cohort of 861 NMD patients.

## 2. Methods

**Figure 1:**
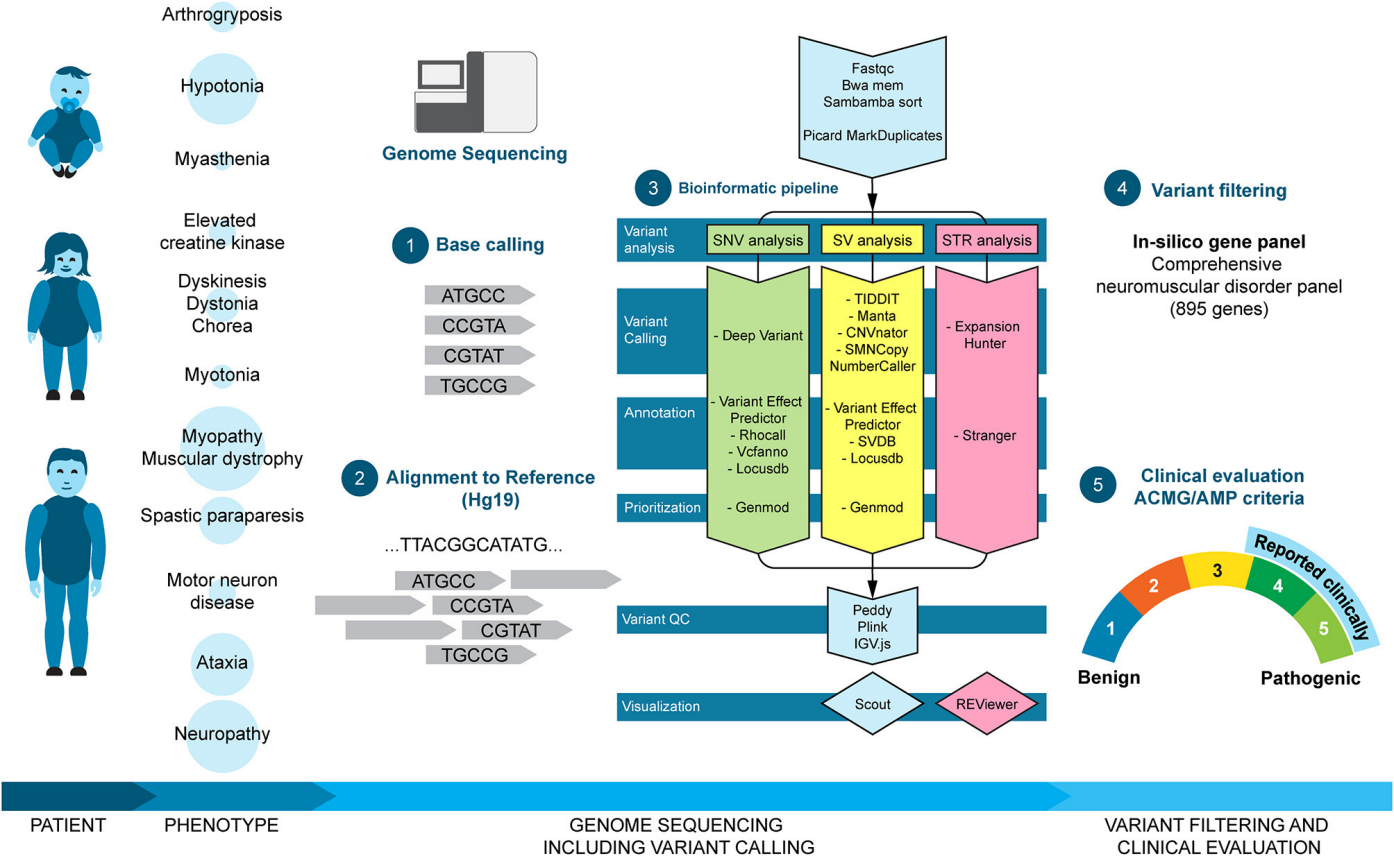
Clinical workflow and bioinformatic pipeline. Patients of different ages were referred for clinical genome sequencing due to neuromuscular disorder phenotypes. The size of the circle behind the phenotype corresponds to the size of the subgroup. Genome sequencing was analyzed using an in-house analysis pipeline including base calling (1), alignment to reference (2), and variant calling and prioritizing (3). Finally, called variants were filtered *in silico* with the NMD gene panel (4) and classified according to the ACMG/AMP criteria (5). Clinically relevant variants (selected class 3, class 4, and class 5) were reported to the referring doctor.

In this retrospective study, genetic results were compiled from all 861 referrals for clinical genome sequencing at our center due to suspected neuromuscular disorders including patients with ataxia and/or spastic paraparesis from January 2016 to October 2022. The cohort included patients of all ages from prenatal cases to elderly patients. Genomic DNA was isolated from whole blood using QIAsymphony (QIAGEN, Hilden, Germany) and the QIAsymphony DSP DNA Midi Kit (cat. no. 937255, QIAGEN, Hilden, Germany) according to manufacturer's protocol. For the prenatal cases, DNA was extracted from a chorion villi biopsy or fetal tissue using the EZ1 Advanced XL instrument (QIAGEN, Hilden, Germany) and the EZ1 DNA Tissue Kit (cat. no. 953034, QIAGEN, Hilden, Germany) following manufacturer's protocol. Individuals that were analyzed clinically before December 2019 (*n* = 455) were previously presented by Stranneheim et al. (13). Over the years, the number of genes in the NMD panel was updated regularly. Furthermore, bioinformatic pipelines were continuously improved (13). Therefore, prior to compiling the results, all negative cases were reanalyzed with the latest NMD gene panel and bioinformatic pipeline as outlined in Figure 1 and described below.

### 2.1. Genome sequencing

The workflow of clinical genome sequencing at our center has previously been described in detail (13). For all cases, sequencing reads were mapped to GRCh37 (hg19), and patients were analyzed as singletons. In short, we used an in-house developed analysis pipeline (MIP; https://github.com/Clinical-Genomics/MIP) to detect SNVs, INDELs, and SVs (deletions/duplications and balanced aberrations) in a comprehensive panel of known NMD genes (*n* = 895) and known pathogenic STR loci (*n* = 28) (Supplementary Table 1). The panel also included genes associated with ataxia and spastic paraparesis because of a phenotypic overlap and potential clinical difficulty to differentiate between these disorders and pure NMDs. Variants were ranked and visualized in an in-house analysis software (Scout; https://github.com/Clinical-Genomics/scout) (13). Larger SVs (>50 Kb) were visualized in the Cytosure Interpret Software (Oxford Gene Technology) after conversion with a custom program (vcf2cytosure; https://github.com/NBISweden/vcf2cytosure), as described previously (14). SNVs, INDELs, deletions and duplications, and STRs were classified according to the American College of Medical Genetics and Genomics (ACMG)/Association for Molecular Pathology (AMP) guidelines (15) as described previously (16). Class 4 and 5 variants were reported to the referring doctor and are termed as “pathogenic variants.” Moreover, selected class 3 variants, variants of uncertain significance (VUS), were reported either due to a strong and specific overlap between the gene and the patient phenotype or due to compound heterozygosity with a pathogenic variant. STR expansions indicated above normal size (intermediate or pathogenic) by Stranger (https://github.com/Clinical-Genomics/stranger) were inspected using integrative genomics viewer (IGV) for false positive calls. Calls with suspicion of pathogenicity were verified by an alternate method (see Section 2.2). Finally, negative cases with an unspecific or complex phenotype (e.g., hypotonia or additional non-NMD symptoms) and/or a strong suspicion of a genetic disorder (e.g., positive family history) were also screened for known pathogenic variants in a panel including all OMIM disease genes, and reported to the referring doctor, if there was a match between the variant and the patient phenotype (Figure 1). The results were also discussed with the referring doctors, neuropathologists, and neurophysiologists at regular multidisciplinary meetings.

### 2.2. Verification of variants identified by genome sequencing

STR expansions detected in the WGS data were verified with a secondary method, either in-house or at an external lab. In-house validated PCR with electrophoresis and a repeat-primed assay (RP-PCR) were used for *AR, ATXN2, HTT*, and *RFC1*. Confirmation analysis in-house using commercial kits from Asuragen was performed for *C9orf72* (AmplideX^®^ PCR/CE C9orf72 Kit, Asuragen) and *DMPK* (AmplideX^®^ PCR/CE DMPK Kit, Asuragen). Samples with detected expansions in *ATXN7, ATXN8OS, CACNA1A, CNBP, FXN*, or *PABPN1* were sent to an external lab for PCR with electrophoresis and a repeat-primed assay (RP-PCR) analysis (CENTOGENE, Rostock, Germany).

Small (< 50 Kb) deletions and duplications were verified by MLPA or sequencing (with breakpoint PCR). Large (>50 Kb) deletions and duplications were verified by chromosomal microarray, using a custom 1,80,000 oligonucleotide array, designed with an even distribution across the genome, with ~18 Kb probe spacing (AMADID:031035, Oxford Gene Technology, Begbroke, Oxfordshire, UK). For one individual (case 420), a custom slide with 1 × 1 M oligonucleotides, where probes are designed to cover the exons of 4,645 known disease-causing genes was used (AMADID:068073, Oxford Gene Technology, Begbroke, Oxfordshire, UK). Experiments were performed according to manufacturer's protocol, with some minor changes (14). SNVs with low-quality parameters as well as INDELS were verified by conventional PCR and Sanger sequencing.

### 2.3. Ethical approval

Ethical approval was given by the Regional Ethical Review Authority in Stockholm (ethics permit numbers KS 2012/222-31-3, addendum 1 2023-00178-02). The permit allows for clinical samples to be used for the analysis of scientific importance for clinical development. The approval does not require us to obtain written consent for clinical testing. The research followed the principles of the Helsinki Declaration.

## 3. Results

### 3.1. Description of the cohort

**Figure 2:**
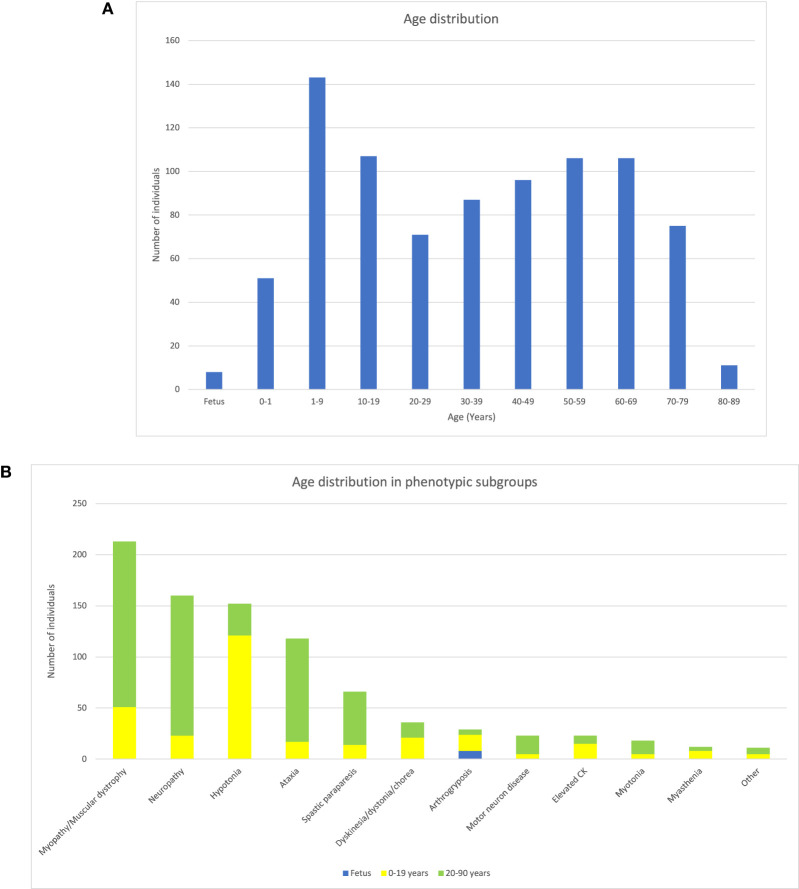
Description of the cohort of NMD patients. **(A)** Bar chart showing the distribution of patient ages. **(B)** Bar chart showing the distribution of phenotype subgroups as well as the relation between prenatal cases, pediatric patients, and adult patients in each phenotype group. Patients 0–19 years of age (yellow), ≥20–90 years of age (green), and prenatal cases (blue).

The cohort included 861 patients (472 male and 381 female subjects) between the age of 1 day and 88 years (median age 35 years), as well as eight prenatal cases. Pediatric patients (< 20 years) accounted for one-third of the cases. The distribution of age is shown in Figure 2A. All patients were referred for genome sequencing due to clinical findings compatible with a neuromuscular disorder. Based on clinical findings, the cohort was divided into eleven phenotypic subgroups (myopathy/muscle dystrophy, neuropathy, ataxia, myasthenia, asymptomatic elevation of creatine kinase, unspecified hypotonia, dyskinesia/dystonia/chorea, spastic paraparesis, motor neuron disease, myotonia, and arthrogryposis). The subgroup “motor neuron disease” includes patients with spinal muscular atrophy (SMA) and similar disorders, while it excludes those with a diagnosis of amyotrophic lateral sclerosis. Overall, the most common phenotypic subgroup was myopathy/muscular dystrophy (215/861, 25%). However, among pediatric patients, the most common phenotype was unspecified hypotonia (119/301, 40%). The prevalence of all phenotypes, as well as the relative distribution in pediatric and adult patients, is shown in Figure 2B.

### 3.2. Diagnostic yield

Genome sequencing and analysis of SNV/INDELs in the 895 gene panel detected pathogenic or likely pathogenic variants in 161 out of 861 patients (19%) (Supplementary Table 2). An additional 81 patients (9%) (Supplementary Table 3) harbored variants that were classified as VUSs, while they were still regarded as strong candidates for being causative of the patient's phenotype. The majority of these VUSs were ultra-rare missense variants that were predicted to be damaging and located in genes with a known association with a specific phenotype that matched the patient findings. For clarity, those VUSs are not included when calculating the diagnostic yield.

The genome sequencing data were also analyzed regarding STRs at 28 loci (Supplementary Table 1), with pathogenic repeat expansions detected in 35/861 patients (4%) (Table 1). In these patients, the most prevalent diagnosis was cerebellar ataxia, neuropathy, and vestibular areflexia syndrome (CANVAS, OMIM #614575) due to bi-allelic expansions in *RFC1* (13 individuals). Among all individuals with repeat expansions, ataxia was the most common phenotypic finding. However, pathogenic expansions were detected in all phenotypic subgroups, except myasthenia and isolated CK-elevation. Only one pathogenic repeat expansion was found in the pediatric cohort, a 1-year-old girl with progressive hypotonia harbored a pathogenic CAG-repeat in *ATXN7*, compatible with spinocerebellar ataxia 7 (SCA7, OMIM #164500).

**Table 1:** Patients with pathogenic expansions of known STR loci.

**Patient**	**Age group**	**Phenotype group**	**Gene**	**Nucleotides in repeat (pathogenic repeat)**	**Zygosity**	**Diagnosis (MIM ID)**	**Inheritance pattern**	**Heredity**
384	Adult	Motorneuron disease						Inherited
419	Adult	Myopathy/ muscular dystrophy	*AR*	CAG	Hemi	Spinal and bulbar muscular atrophy of Kennedy (MIM#313202)	XL	NI
584	Adult	Myopathy/ muscular dystrophy						NI
516	Adult	Ataxia	*ATXN2*	CAG	Het	Spinocerebellar ataxia 2 (MIM#183090)	AD	NI
326	Pediatric	Hypotonia	*ATXN7*	CAG	Het	Spinocerebellar ataxia 7 (MIM#164500)	AD	Inherited (anticipation)
247	Adult	Ataxia						NI
678	Adult	Ataxia	*ATXN8OS*	CAG	Het	Spinocerebellar ataxia 8 (MIM#608768)	AD	NI
693	Adult	Ataxia						NI
394	Adult	Spastic paraplegia						NI
317	Adult	Myopathy/ muscular dystrophy	*C9orf72*	GGGGCC	Het	Frontotemporal dementia and/or amyotrophic lateral sclerosis 1 (MIM#105550)	AD	NI
788	Adult	Ataxia						NI
612	Adult	Ataxia	*CACNA1A*	CAG	Het	Episodic ataxia, type 2 (MIM#108500)	AD	NI
165	Adult	Myotonia						Inherited
323	Adult	Spastic paraplegia	*CNBP*	CCTG	Het	Myotonic dystrophy 2 (MIM#602668)	AD	NI
170	Adult	Arthrogryposis						Inherited (anticipation)
572	Adult	Myopathy/ muscular dystrophy	*DMPK*	CTG	Het	Myotonic dystrophy 1 (MIM#160900)	AD	NI
628	Adult	Myopathy/ muscular dystrophy						NI
64	Adult	Neuropathy	*FXN*	GAA	Hom	Friedreich ataxia (MIM#229300)	AR	NI
613	Adult	Neuropathy						NI
411	Adult	Dyskinesia/ dystonia/chorea	*HTT*	CAG	Het	Huntington disease (MIM#143100)	AD	NI
359	Adult	Hypotonia						NI
734	Adult	Myopathy/ muscular dystrophy	*PABPN1*	CGN	Het	Oculopharyngeal muscular dystrophy (MIM#164300)	AD	NI
101	Adult	Ataxia						NI
120	Adult	Ataxia						NI
248	Adult	Ataxia						NI
301	Adult	Ataxia						NI
372	Adult	Ataxia						NI
415	Adult	Ataxia	*RFC1*	AAAGG (AAGGG)	Hom	Cerebellar ataxia, neuropathy, and vestibular areflexia syndrome, CANVAS (MIM#614575)	AR	NI
462	Adult	Ataxia						NI
652	Adult	Ataxia						NI
765	Adult	Ataxia						NI
820	Adult	Ataxia						NI
83	Adult	Neuropathy						NI
388	Adult	Neuropathy						NI
710	Adult	Neuropathy						NI

AD, autosomal dominant; AR, autosomal recessive; Hemi, hemizygous; Het, heterozygous; Hom, homozygous; NI, not investigated; XL, X-linked.

**Figure 3:**
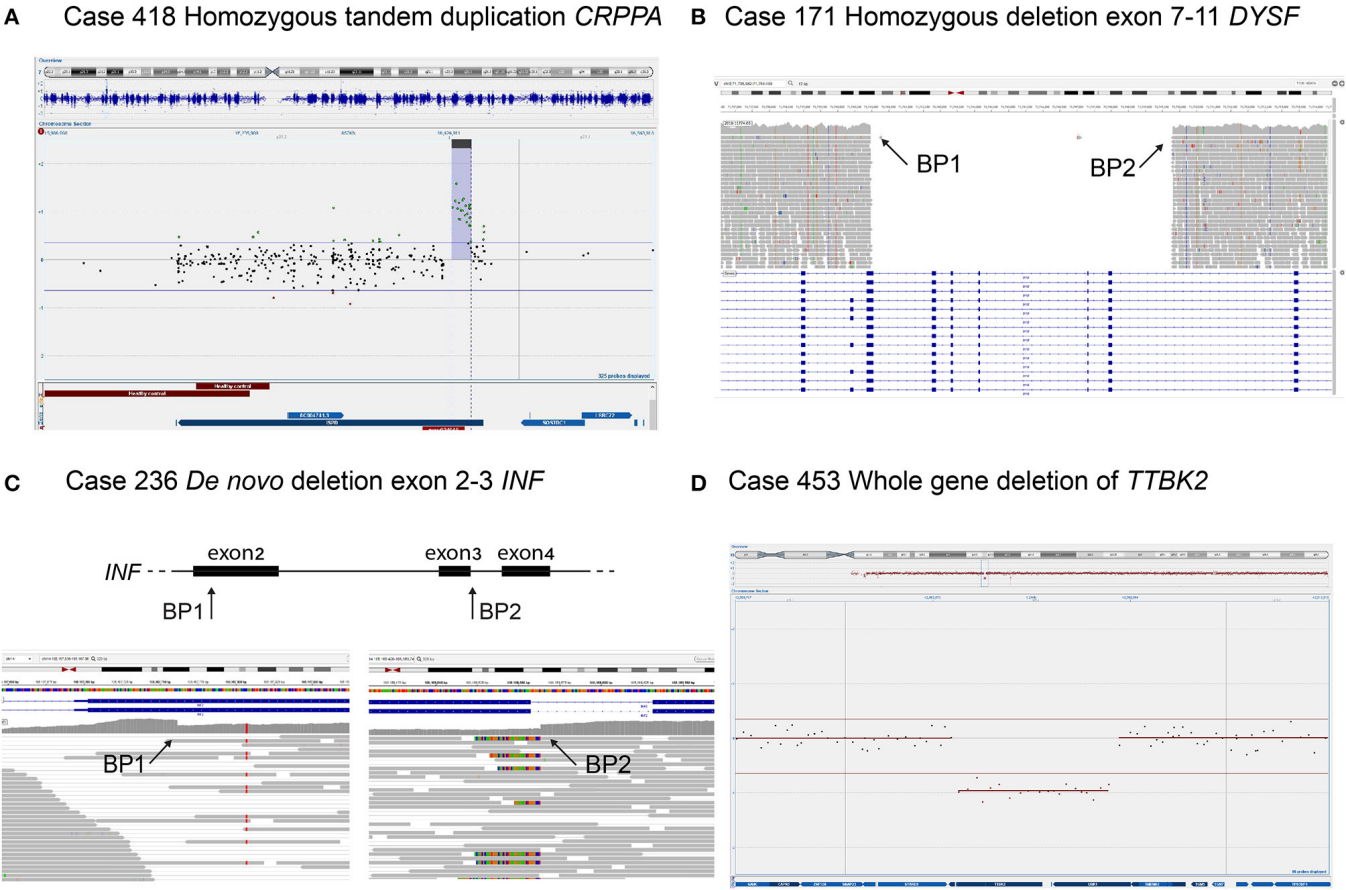
Four individuals with SVs detected in NMD genes. **(A)** Homozygous tandem duplication of *CRRPA* in an individual with muscular dystrophy, CK 90, and mild ID. Duplication is shown in copy number variant (CNV) plot from chromosomal microarray with a 1M oligonucleotide slide. **(B)** Homozygous deletion of exon 7–11 in *DYSF* in an individual with limb girdle muscular dystrophy and disease onset in their twenties. The deletion is shown in the integrative genomics viewer (IGV) where there are no reads (gray arrows) mapping to the deleted region. **(C)** *De novo* deletion of exon 2–3 in *INF2* in an individual with the onset of sensory motor neuropathy in early childhood and renal failure before the age of 20 years. The heterozygous deletion is shown in IGV displaying the two breakpoints with a sudden drop and a rise in coverage (arrows), respectively. **(D)** Whole-gene deletion of *TTBK2* in an individual with the onset of ataxia around 40 years of age and sensory-motor neuropathy at nearly 50 years of age. Deletion is shown in CNV plot from chromosomal microarray.

The analysis of structural variants in the NMD gene panel, including deletion of *SMN1* and deletion/duplication of *PMP22*, identified pathogenic SVs in 36 patients (4%) (Table 2) (sizes ranging from 1.8 Kb to 18.8 Mb). The majority of the variants were intragenic deletions or duplications in *DMD* (11 individuals). Interestingly, seven of them were female patients with isolated CK-elevation or symptoms such as muscle pain and mild muscle weakness. The second most common gene with pathogenic findings detected by SV-analysis was *PMP22*, seen in eight patients with Charcot–Marie-Tooth type 1A (CMT1A, OMIM #118220) and one patient with neuropathy, hereditary, with liability to pressure palsies (HNPP, OMIM #162500). Notably, the majority of the CMT1A patients were children. All of them had an affected parent without a previous genetic diagnosis either due to mild symptoms or migration from countries without advanced healthcare. Homozygous deletion of *SMN1* was seen in two individuals; a 1-year-old boy and a 52-year-old male, both presented with hypotonia. The number of *SMN2* copies in these patients was three and four, respectively, and the phenotypes were classified as SMA type 2 and 4. There were no cases with SMA type 1, likely reflecting that these patients are recognized clinically and confirmed with targeted testing of *SMN1*, rather than referred for genome sequencing. In total, seven patients harbored rare, non-recurrent SVs in single genes, including *CRRPA, DYSF, INF2*, and *TTBK2* presented in Figure 3. Large SVs, involving several genes, were identified in seven individuals (excluding *PMP22*) (Table 2).

**Table 2:** Patients with pathogenic and likely pathogenic SVs.

	**Patient**	**Age group**	**Phenotype subgroup**	**Gene**	**Variant type**	**Cytoband**	**Aberration (Genome build Hg19)**	**Zygosity**	**Size**	**Diagnosis (OMIM ID)**	**Inheritance pattern**	**Heredity**
Small structural variants (single gene)	849	Pediatric	Hypotonia	*ATP8A2*	Del	13q12.13	chr13:26,207,191-26,348,939del	Hom	142 Kb	Cerebellar ataxia, mental retardation, and dysequilibrium syndrome 4 (OMIM #615268)	AR	Parents heterozygous carriers
	418	Adult	Myopathy/muscular dystrophy	*CRPPA*	Dup	7p21.2	chr7:16,427,126-16,447,058trp	Hom	20 Kb	Muscular dystrophy-dystroglycanopathy, type A (OMIM #614643)	AR	NI
	13	Adult	Myopathy/muscular dystrophy				chrX:31,973,924-32,017,000del	Het	43 Kb			*De novo*
	234	Pediatric	Myopathy/muscular dystrophy				chrX:32,834,322-32,947,628del	Hem	113Kb			Inhertited (*de novo* in parent)
	522	Pediatric	Myopathy/muscular dystrophy				chrX:31,840,023-32,065,091del	Hem	255Kb			*De novo*
	631	Adult	Myopathy/muscular dystrophy				chrX:31,782,757-31,815,273del	Het	33Kb			*De novo*
	779	Adult	Myopathy/muscular dystrophy				chrX:32,232,439-32,277,030delinsTT	Het	45Kb			NI
	135	Pediatric	Elevated CK	*DMD*	Del	Xp21.2	chrX:32,257,014-32,408,862del	Het	152Kb	Duchenne muscular dystrophy (OMIM #310200)	XL	NI
	757	Pediatric	Elevated CK				chrX:31,939,224-31,960,909del	Becker muscular dystrophy (OMIM #300376)		Het	22Kb	NI
	491	Pediatric	Hypotonia				chrX:31,588,218-31,902,422del	Hem	314Kb			Inherited (*de novo* in parent)
	687	Pediatric	Hypotonia				chrX:31,665,925-31,903,635del	Hem	238Kb			Inherited
	832	Pediatric	Hypotonia				chrX:31,774,297-31,902,368del	Het	128Kb			*De novo*
	351	Adult	Elevated CK			Dup	chrX:32,911,288-33,108,882dup	Het	198Kb			NI
	171	Adult	Myopathy/muscular dystrophy	*DYSF*	Del	2p13.2	chr2:71,740,967-71,749,805del	Hom	8.8 Kb	Muscular dystrophy, limb-girdle, autosomal recessive 2 (OMIM #253601)	AR	NI
	236	Adult	Neuropathy	*INF2*	Del	14q32.33	Chr14:105,167,761-105,169,563del	Het	1.8 Kb	Charcot-Marie-Tooth disease, dominant intermediate E (OMIM #614455) Glomerulosclerosis, focal segmental, 5 (OMIM #613237)	AD	NI
	57	Pediatric	Myopathy/muscular dystrophy	*LAMA2*	Dup	6q22.33	chr6:129,655,050-129,670,160dup	Hom	15 Kb	Muscular dystrophy, congenital, merosin deficient or partially deficient (OMIM #607855)	AR	Parents heterozygous carriers
	494	Pediatric	Hypotonia	*PAFAH1B1*	Del	17p13.3	chr17:2,575,563-2,579,050del	Het	3.5 Kb	Lissencephaly 1 (OMIM #607432)	AD	*De novo*
	644	Pediatric	Hypotonia	*SMN1*	Del	5q13.2	whole gene	Hom	NI	Spinal muscular atrophy-1 (OMIM #253300)	AR	Parents heterozygous carriers
	822	Adult	Hypotonia		Del			Hom				NI
	453	Adult	Neuropathy	*TTBK2*	Del	15q15.2	chr15:43,029,580-43,373,496del	Het	344 Kb	Spinocerebellar ataxia 11 (OMIM #604432)	AD	NI
Large structural variants (multiple genes)	478	Adult	Neuropathy	*CAMTA1 RERE*	Del	1p36.31-36.23	chr1:5,415,942-8,891,221del	Het	3.5 Mb	Cerebellar dysfunction with variable cognitive and behavioral abnormalities (OMIM #614756) Neurodevelopmental disorder with or without anomalies of the brain, eye, or heart (OMIM #616975)	AD	NI
	379	Pediatric	Hypotonia		Del	2q33.3-q36.1	chr2:207,110,692-222,914,109del	Het	15.8 Mb	NA	AD	*De novo*
	753	Pediatric	Hypotonia		Del	9p24.3-p23	chr9:204,178-11,884,611del	Het	11.7 Mb	9p24 deletion syndrome	AD	NI
	219	Pediatric	Myopathy/muscular dystrophy		Del	14q13.3-q21.2	chr14:36,843,519-45,031,176del	Het	8.2 Mb	NA	AD	*De novo*
	629	Pediatric	Hypotonia		Del	15q11.2q13.1	chr15:22,747,001-28,660,000	Het	5.8 Mb	Prader-Willi syndrome (OMIM #176270)	AD	NI
	793	Pediatric	Dyskinesia/dystonia/ chorea		Dup	16p11.2	chr16:29,447,844-30,199,168dup	Het	751 Kb	16p11.2 duplication syndrome	AD	Inherited (incomplete penetrance)
	124	Adult	Neuropathy				chr17:14,070,993-15,477,002dup	Het	1.4 Mb			NI
	364	Pediatric	Neuropathy	PMP22	Dup	17p12	chr17:14,089,001-15,486,058dup	Het	1.4Mb	Charcot-Marie-Tooth disease, type 1A (OMIM#118220)	AD	Inherited (symptomatic parent)
	441	Pediatric	Neuropathy				chr17:14,192,001-15,486,000dup	Het	1.3Mb			De novo
	451	Pediatric	Neuropathy				chr17:14,192,001-15,303,000dup	Het	1.1Mb			NI
	649	Adult	Neuropathy				chr17:14,141,001-15,487,000dup	Het	1.3 Mb			NI
	771	Adult	Neuropathy	*PMP22*	Dup	17p12	chr17:14,192,001-15,250,000dup	Het	1.1Mb	Charcot-Marie-Tooth disease, type 1A (OMIM#118220)	AD	NI
	847	Pediatric	Neuropathy				chr17:14,192,001-15,486,000dup	Het	1.3Mb			NI
	509	Pediatric	Hypotonia				chr17:14,092,001-15,494,000dup	Het	1.4Mb			De novo
	589^*^	Adult	Neuropathy	*PMP22*	Del	17p12	chr17:14,090,001-15,477,000del	Het	1.4 Mb	Polyneuropathy, recurrent, with pressure palsies (OMIM #162500)	AD	NI
	289	Pediatric	Hypotonia	*PLP1*	Dup	Xq22.1-q22.3	chrX:100,351,605-106,073,360dup	Hem	5.6 Mb	Pelizaeus-Merzbacher disease (OMIM #312080)	XL	NI

* Also diagnosed with heterozygous missense variant in MPZ (NM_000530.8:c.418T>A) causing Charcot-Marie-Tooth 1B.

AD, autosomal dominant; AR, autosomal recessive; CK, creatine kinase; Del, deletion; Dup, duplication; Hem, hemizygous; Het, heterozygous; Hom, homozygous; Kb, kilo bases; Mb, mega bases; NA, not applicable; NI, not investigated; XL, X-linked.

Finally, genome-wide screening for pathogenic variants affecting genes not included in the NMD gene panel resulted in diagnostic findings in an additional 18 patients (Supplementary Table 4). Most patients in this group had early-onset hypotonia and, over time, the majority developed additional symptoms such as intellectual disability (ID) or epilepsy (EP). A recurrent diagnosis in this group was Coffin–Siris syndrome (CSS1, OMIM #135900; CSS2, OMIM #614607) (four individuals). Patients with pathogenic variants in genes outside the NMD gene panel are not included when calculating the diagnostic yield.

**Figure 4:**
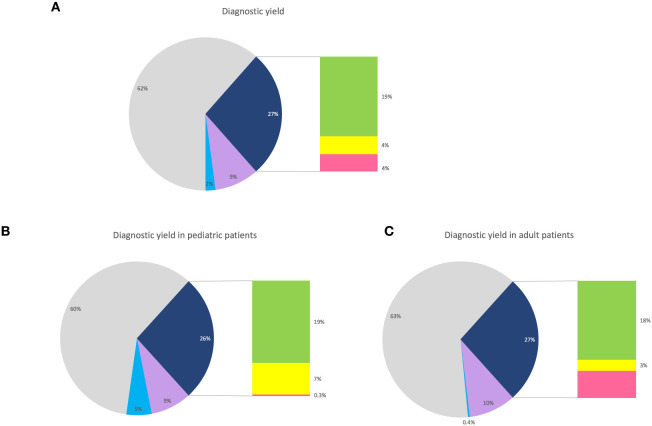
Diagnostic yield. **(A)** Pie chart showing the percentage of all patients with pathogenic or likely pathogenic findings in NMD genes (dark blue), with a bar chart showing the distribution of SNVs/INDELs (green), SVs (yellow), and STRs (pink). For visualization, patients with VUS in NMD genes (purple) and pathogenic findings in non-NMD genes (light blue). **(B)** Pie chart showing the percentage of pediatric patients with pathogenic or likely pathogenic findings in NMD genes (dark blue), with a bar chart showing the distribution of SNVs/INDELs (green), SVs (yellow), and STRs (pink). For visualization, patients with VUS in NMD genes (purple) and pathogenic findings in non-NMD genes (light blue). **(C)** Pie chart showing the percentage of adult patients with pathogenic or likely pathogenic findings in NMD genes (dark blue), with a bar chart showing the distribution of SNVs/INDELs (green), SVs (yellow), and STRs (pink). For visualization, patients with VUS in NMD genes (purple) and pathogenic findings in non-NMD genes (light blue).

Altogether, the diagnostic yield increased from 19% (161/861 patients), with SNV/INDEL analysis in the NMD gene panel, to 27% (232/861 patients) with the addition of STR- and SV-analyses (Figure 4A). The total diagnostic yield was the same in pediatric patients compared to adults. However, the prevalence of pathogenic SVs and STRs differed between the groups, with STRs being more frequent among adult patients, while SVs were more prevalent among pediatric patients (Figures 4B, C).

**Figure 5:**
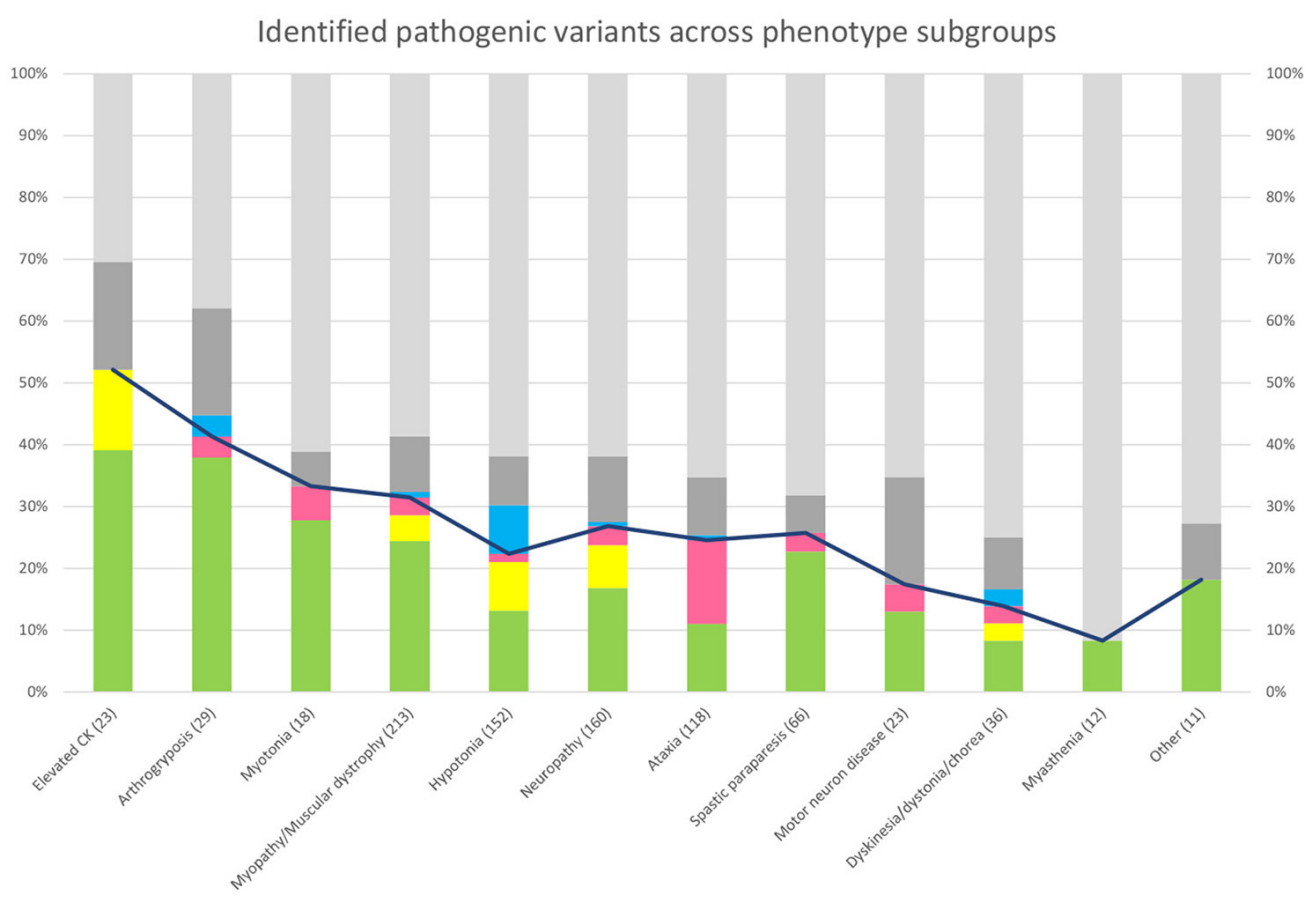
Genetic findings across phenotype subgroups. Bar chart showing the percentage of patients with pathogenic or likely pathogenic SNVs/INDELs (green), SVs (yellow), and STRs (pink). For visualization, patients with pathogenic findings in non-NMD genes (blue) and VUS in NMD genes (dark gray). Line chart showing the diagnostic yield in each phenotypic subgroup.

In the phenotypic subgroups, the highest overall yield was seen in those with asymptomatic elevation of creatine kinase (12/23, 52%) and those with arthrogryposis (12/30, 40%) (Figure 5). Looking at the yield added by STR- and SV-analysis, in the different phenotype groups, the largest increase in yield by STR-analysis was seen in those with ataxia, while the largest increase in yield by SV-analysis was seen in those with hypotonia (Figure 5).

Despite the proven heterogeneity among NMDs, a few genes were recurrently affected in our cohort: *DMD* (*n* = 14), *RFC1* (*n* = 13), *RYR1* (*n* = 9), *PMP22* (*n* = 9), *FKRP* (*n* = 8), *CAP*N*3* (*n* = 7), *COL6A1* (*n* = 7), and *DYSF* (*n* = 5). In total, causative variants were detected in 107 genes (among the 232 patients with pathogenic findings) in the NMD gene panel (Supplementary Table 2).

For all patients in the cohort with an established molecular diagnosis, the most common inheritance pattern was autosomal recessive (45%), followed by autosomal dominant (43%) and X-linked (11%). The remaining 1% of diagnoses had a digenic or mitochondrial inheritance pattern. In pediatric patients, inheritance from the parents was examined. Parental samples were analyzed for 35 of the 50 patients harboring pathogenic variants in genes associated with autosomal dominant disorders. This resulted in 26 variants found to be *de novo*, and nine were inherited from either a healthy parent (*n* = 4) or a symptomatic parent (*n* = 5). The variants that were inherited from a healthy parent were all associated with disorders known to have reduced penetrance or anticipation. For patients with bi-allelic autosomal recessive variants, 23 of the 32 families were followed up, revealing 21 individuals with both parents being healthy heterozygous carriers. For the additional two individuals, one carried a paternally inherited variant in combination with a *de novo* variant, and one individual had a father affected by the same disorder as the child and compound heterozygous for the same variants in *ANO5* as the child. In the latter case, the mother carried a relatively common founder mutation in *ANO5*. The parents were unrelated. For the variants with X-linked inheritance, maternal samples for 12 of 14 variants were investigated, eight were found to be *de novo*, and four inherited from the mother (Table 2; Supplementary Table 2). The individuals who inherited the variant were all boys and thereby hemizygous for the pathogenic variant.

## 4. Discussion

In this study, 861 patients with neuromuscular disorders were analyzed with genome sequencing and comprehensive variant calling. The overall diagnostic yield for pathogenic or likely pathogenic variants was 27% (232/861 patients). Previous studies have shown a diagnostic yield for SNVs/INDELs in NMD patients, varying between 13 and 53% (3–10). One explanation for the variable figures is differences in calculating yield. In our study, we report only pathogenic and likely pathogenic variants according to ACMG classification (15), while many studies with higher yield have included variants of unknown significance. Furthermore, inclusion criteria affect outcome. In the present study, inclusion criteria were set to reflect the clinical landscape, with the inclusion of all patients referred for genome sequencing. This may have affected outcome as not all patients had an a priori strong suspicion of a genetic disorder.

Remarkably, out of all pathogenic and likely pathogenic NMD variants in our cohort, one-third of them were repeat expansions or deletions/duplications.

By applying STR- and SV- callers to the genome sequencing data, 71 patients received a molecular diagnosis. Some of these patients might have reached an etiological diagnosis through subsequent targeted genetic tests. However, requests for targeted tests are often based on thorough clinical investigations, such as muscle biopsies, neurophysiological assessments, and MRI of muscles and/or the CNS. This approach is cost- and time-consuming, and it may lead to a delay in a definite diagnosis for the patient. Furthermore, for most of the patients with pathogenic STRs and SVs, the specific diagnosis was difficult to pinpoint prior to genetic testing despite a meticulous clinical workup.

In our cohort, as described above, the major diagnostic advantage of genome sequencing over targeted gene panels or exome sequencing was the potential of adding bioinformatic tools for STR- and SV-analysis. In addition, genome sequencing offered the possibility to analyze deep intronic variants, exemplified by two *de novo* variants in *COL6A1* (c.930 + 189C > T) and one homozygous variant in *DYSF* (c.5785-824C > T), detected in the cohort. Moreover, genome sequencing (like exome sequencing) also offers the possibility to analyze genes not included in the primary gene list. The NMD gene list applied in this study is a broad panel based on the expanded definition of neuromuscular disorders to include also genes associated with ataxia and spastic paraparesis. Despite using a comprehensive gene list, a fraction of the patients with a presumed neuromuscular disorder were diagnosed with genetic disorders due to variants in genes outside the panel. The vast majority of these patients were children with hypotonia or movement disorders, in whom pathogenic variants in genes associated with ID- or EP-syndromes were detected. These results illustrate the difficulty in applying gene panels based on disease groups and favor an even broader gene panel, particularly for children with unspecific phenotypes such as hypotonia.

In summary, this study of genome sequencing and comprehensive variant calling in 861 patients with NMDs is the largest study to date. The main finding of the study shows that one-third of the patients with pathogenic findings in the NMD gene panel have a repeat expansion or a deletion/duplication as the cause of their phenotype. This finding leads us to conclude that it is crucial to include bioinformatic pipelines for STR- and SV-analysis in massive parallel sequencing diagnostics for patients with neuromuscular disorders.

## Data availability statement

The datasets presented in this article are not readily available because of ethical and privacy restrictions. Requests to access the datasets should be directed to the corresponding author.

## Ethics statement

The studies involving human participants were reviewed and approved by the Regional Ethical Review Authority in Stockholm, Sweden. Written informed consent from the participants' legal guardian/next of kin was not required to participate in this study in accordance with the national legislation and the institutional requirements.

## Author contributions

Conceptualizing was done by AL, MK, and TS. The contribution of samples and phenotyping was done by GS, HHj, IN, KN, MEn, MPa, RP, and SA. Data curation was done by AL, AH, B-MA, DN, HM, HT, HHe, MEk, MK, and MPe. Formal analysis was done by AL, MEk, and MK. Funding acquisition and project administration was done by AL and MK. Visualization and writing of the first draft were done by MEk and MK. Writing—review and editing were done by AL, AH, B-MA, DN, GS, HM, HT, HHe, HHj, IN, KN, MEn, MEk, MK, MPa, MPe, RP, SA, and TS. All authors contributed to the article and approved the submitted version.
